# Supplementary material for: Association between oral microbiome and seven types of cancers in East Asian population: a two-sample Mendelian randomization analysis
Source: Front Mol Biosci. 2023 Nov 21;10:1327893. doi: 10.3389/fmolb.2023.1327893 (PMC10702768; doi:10.3389/fmolb.2023.1327893)
Supplement: Supplementary file 1 [file DataSheet1.ZIP › Supplementary Materials/MR plots for tongue/tongue═╝/Prostate cancer/pheno.796_to_prostate cancer_forest.pdf]

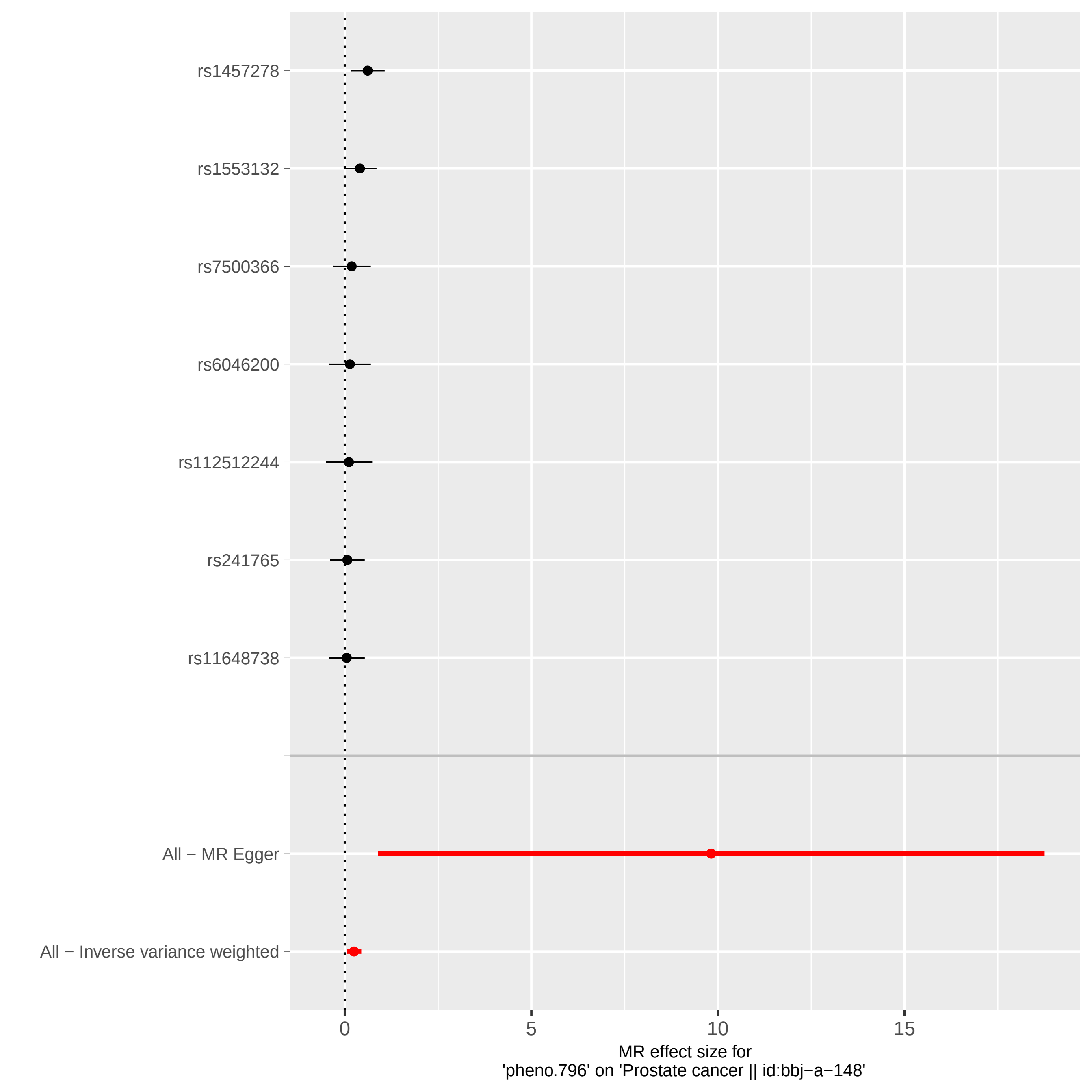

rs1457278
rs1553132
rs7500366
rs6046200
rs112512244
rs241765
rs11648738
All − MR Egger
All − Inverse variance weighted
0
5
10
15
MR effect size for
'pheno.796' on 'Prostate cancer || id:bbj−a−148'
